# Pediatric perforated appendicitis diagnosis based on the C-reactive protein/prealbumin ratio

**DOI:** 10.1038/s41598-024-55108-3

**Published:** 2024-03-20

**Authors:** Junshan Long, Jing Zhang, Gong Chen, Xiaoxia Su, Baowei Qiu, Qi Dong

**Affiliations:** 1 Department of General Surgery, Hainan Women and Children’s Medical Center, Changbin Road, Haikou, Hainan China; 2 Department of General Surgery, Children’s Hospital of Fudan University, Shanghai, China

**Keywords:** Perforated appendicitis, Predictor, Pediatric, C-reactive protein, Prealbumin, Odds ratio, Biomarker, Early intervention, Diagnostic markers, Biomarkers, Risk factors

## Abstract

Pediatric perforated appendicitis, prone to multiple complications, necessitates identifying potential serum biomarkers for early diagnosis and intervention. A cross-sectional study was conducted on patients under 16 with acute appendicitis, admitted to Hainan Women and Children’s Medical Center from January 2019 to July 2023. The patients were categorized into perforated and non-perforated groups. Among the 313 included patients, 106 (33.87%, 95% CI 28.59–39.14%) developed perforation. The C-reactive protein to prealbumin ratio (CPA) showed a significant difference between the perforated and non-perforated groups [6.63 (2.9–13.02) vs. 0.7 (0.11–2.18), *p* < 0.001]. The AUC of CPA on the ROC curve was 0.691 (95% CI 0.513–0.869, *p* = 0.084) in patients under 4. In patients aged 4–9, the sensitivity of CPA > 3 predicting perforation was 76.2%, with a specificity of 81.6%, and an AUC of 0.816 (95% CI 0.747–0.886, *p* < 0.001). For patients aged 9–16, the sensitivity of CPA > 2.2 predicting perforation was 85%, with a specificity of 85.7%, and an AUC of 0.919 (95% CI 0.859–0.979, *p* < 0.001). CPA > 3 and CPA > 2.2 can predict perforated appendicitis in patients aged 4–9 and 9–16, respectively.

## Introduction

The incidence of perforated appendicitis in children surpasses than in adults^1^. Approximately 20% to 45% of pediatric patients diagnosed with appendicitis progress to perforated appendicitis^2,3^. The occurrence of perforated appendicitis can result in intra-abdominal abscesses, diffuse peritonitis, sepsis, and other complications, leading to prolonged hospitalization and a substantial increase in medical costs^4,5^. Thus, identifying reliable markers for early diagnosis of perforated appendicitis holds significant clinical importance.

While the severity of acute appendicitis can be evaluated through abdominal ultrasound and computed tomography scans, accessibility to these tests may be limited in primary care settings. Challenges such as the need for sedation in some pediatric cases, unavailability of technicians to generate final reports, or a lack of qualified personnel to perform these tests might hinder access^6,7^. Consequently, serum biomarkers have gained attention as potential for disease prediction and diagnosis in recent years. Research has highlighted several markers with predictive value for pediatric perforated appendicitis, including the neutrophil-to-lymphocyte ratio^8^, platelet-to-lymphocyte ratio^9^, elevated serum total bilirubin level^10^, increased C-reactive protein (CRP)^11^, decreased serum sodium levels^12^, and elevated erythrocyte sedimentation rate^13^. This study aims to fill this gap by conducting an evaluation of the CRP to prealbumin ratio (CPA) as a potential marker for pediatric perforated appendicitis.

CRP and prealbumin serve as indicators of inflammation and nutritional status, with prealbumin reflecting recent nutritional conditions and the severity of acute inflammatory diseases^14^. This study aims to investigate the potential clinical significance of CPA as a predictive marker for of perforated appendicitis in children.

## Materials and methods

The study conducted an observational, cross-sectional analysis of data from children diagnosed with acute appendicitis at Hainan Women and Children’s Medical Center between January 2019 and July 2023. Inclusion criteria encompassed patients up to 16 years old with acute appendicitis. Exclusion criteria were defined as follows: (1) patients who did not undergo appendectomy; (2) patients with a pathological diagnosis of chronic appendicitis; (3) patients who received antibiotics before admission.

**Figure 1:**
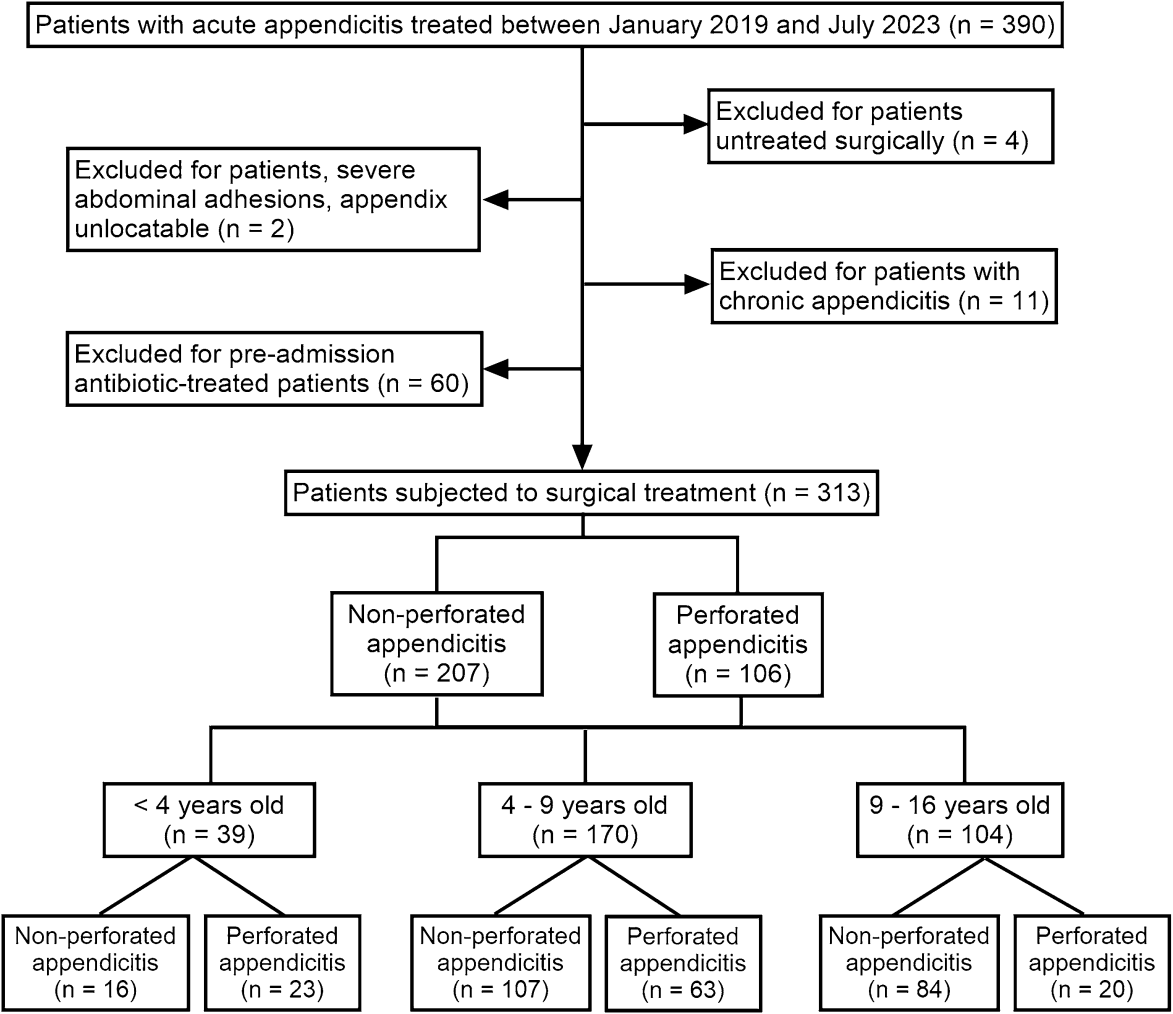
Flowchart of this study. Flowchart of this study, including patient inclusion, exclusion, and grouping.

During the study period, a total of 390 pediatric patients were admitted to the hospital. Out of this cohort, 4 patients did not undergo surgical treatment, 2 had their appendices not located due to severe abdominal adhesions, 11 were diagnosed with chronic appendicitis, and 60 had received antibiotics prior to admission, leading to their exclusion from the study. Consequently, 313 patients were included as research subjects, as outlined in Fig. 1.

### Clinical data

Basic information about the patients, such as age, sex, maximum body temperature before surgery, and duration of symptoms, was extracted from the electronic medical record system. To maintain objectivity, subjective data,including abdominal signs, were not considered. Laboratory test results were collected for each patient, encompassing white blood count (WBC), neutrophil ratio, lymphocyte ratio, hemoglobin, platelets, CRP, procalcitonin, total bilirubin, direct bilirubin, albumin, prealbumin, alanine aminotransferase, aspartate aminotransferase, serum potassium, serum sodium, and serum chlorine. The CPA is determined by dividing the serum CRP by prealbumin. Additionally, the NLR is obtained by dividing the absolute number of neutrophils by the absolute number of lymphocytes, while the PLR is calculated by dividing the absolute number of lymphocytes by the absolute number of platelets.

### Grouping and perforation definitions

Laparoscopic appendectomy is employed in the management of acute appendicitis in pediatric patients. The cohort was stratified into two categories: perforated appendicitis and non-perforated appendicitis, determined by intraoperative observations and pathological findings. The specific criteria characterizing non-perforated appendicitis encompass congestive, purulent, and gangrenous appendicitis without any discernible macroscopic or microscopic perforation. On the other hand, perforated appendicitis is defined as appendicitis featuring either macroscopic or microscopic perforation. Furthermore, the patient cohort was sub-divided into age-specific groups: below 4 years of age, 4–9 years of age, and 9–16 years of age. This subdivision was undertaken to scrutinize the diagnostic efficacy of CPA across distinct age brackets.

### Statistical analysis

Continuous variables are expressed as median and interquartile range (IQR), while categorical variables are presented as counts and percentages. Wilcoxon rank sum tests are applied to continuous variables, and Chi-square tests and Fisher’s exact tests are employed for categorical variables, as deemed appropriate. The Friedman test is utilized to ascertain the significance of differences between perforated appendicitis and non-perforated appendicitis subgroups. For laboratory tests, continuous data undergo binary classification based on the median before univariate analysis. Results exhibiting statistical significance are subjected to multivariate analysis. Logistic regression analysis is employed to identify risk factors for perforated appendicitis. The diagnostic efficacy of CPA in predicting perforated appendicitis is evaluated through receiver operating characteristic (ROC) curve analysis. The area under the curve (AUC) and the 95% confidence interval (CI) are calculated, and the sensitivity and specificity are determined using appropriate cut-off values. All statistical analyses are conducted using SAS 9.4 (SAS Institute, Cary, NC, USA). All tests are two-sided, with *p* < 0.05 considered indicative of a statistically significant difference.

### Ethics declarations

The study received approval from the Ethics Committee of the Hainan Women and Children's Medical Center (HNWCMC MEC NO.102 OF 2023).

### Informed consent

The patient's guardian consented to the use the patient's blood and tissue samples for clinical research and signed a written informed consent.

## Results

**Figure 2:**
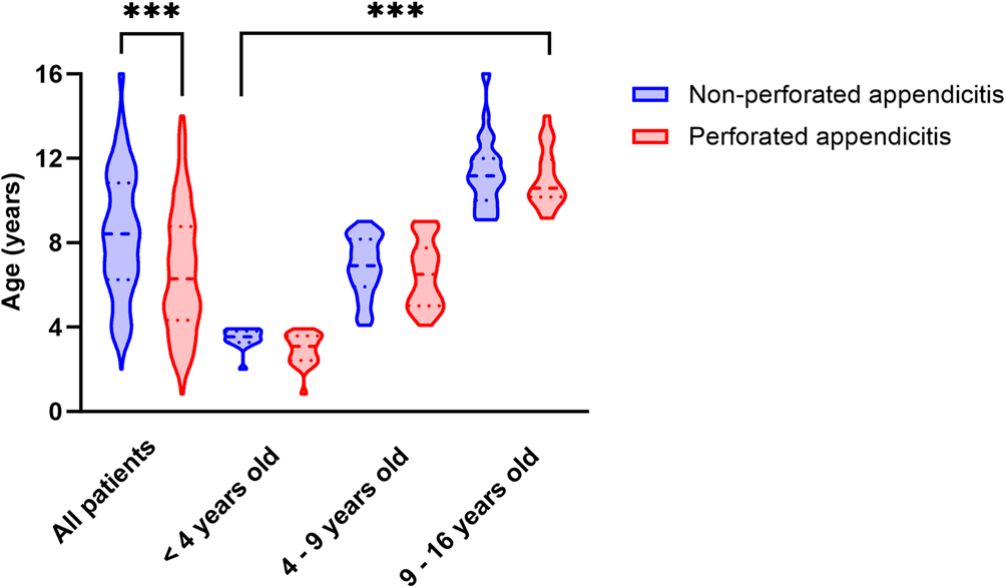
Distribution of non-perforated appendicitis and perforated appendicitis by age group. The distributions of non-perforated appendicitis and perforated appendicitis exhibited significant differences across age groups. In children below 16 years of age, the median age of children with non-perforated appendicitis is 8.42 (6.25–10.83), while the median age of those with perforated appendicitis is 6.29 (4.33–8.75), demonstrating significant differences (*p* < 0.001). The distributions of non-perforated appendicitis and perforated appendicitis varied significantly across age subgroups (*p* < 0.001). ***Significant differences *p* < 0.001.

The study included a total of 313 children diagnosed with acute appendicitis, comprising 184 males (58.79%). Among them, 106 (33.87%, 95% CI 28.59–39.14%) developed perforated appendicitis. There was no significant difference in sex distribution between the perforated and non-perforated appendicitis groups (*p* = 0.126), indicating comparable data. Notably, the distribution of perforated appendicitis varied significantly across different age groups (*p* < 0.001), as illustrated in Fig. 2.

Among the 39 patients below 4 years of age, 23 experienced perforation (58.97%, 95% CI 42.82–75.13%). In the 170 patients aged 4–9 years, perforation occurred in 63 cases (37.06%, 95% CI 29.72–44.39%). Among the 104 patients aged 9 to 16 years, perforation occurred in 20 cases (19.23%, 95% CI 11.53–26.93%). The incidence of perforated appendicitis was notably higher in younger children (*p* < 0.001).

In children under 16 years of age, there were significant differences in the distributions of symptom duration and body temperature between the perforated appendicitis and non-perforated appendicitis groups (*p* < 0.001). Patients with perforated appendicitis were typically diagnosed later (> 72 h) than those with non-perforated appendicitis [52 (49.06%) vs. 39 (18.84%)], and they were more likely to experience high fever (body temperature > 38.5 °C) [57 (53.77%) vs. 36 (17.39%)]. Furthermore, significant variations existed between the perforated and non-perforated appendicitis groups in terms of WBC counts, neutrophils ratio, lymphocytes ratio, CRP, procalcitonin, total bilirubin, direct bilirubin, albumin, prealbumin, and other laboratory test results (*p* < 0.05). Particularly, elevated levels of CRP and procalcitonin in the perforated appendicitis group suggested a more severe inflammatory and infectious state. The CPA in the perforated appendicitis group was significantly higher than compared to the non-perforated appendicitis group [6.63 (2.9–13.02) vs. 0.7 (0.11–2.18), *p* < 0.001]. Similarly, the NLR [8.6 (5.14–14.08) vs. 6.21 (2.83–11.2), *p* < 0.001] and PLR [2.46 (1.56–3.44) vs. 1.72 (1.14–2.85), *p* < 0.001] also exhibited significante differences between the two groups, as detailed in Table 1.

**Table 1:** Baseline data on non-perforated and perforated appendicitis.

Characteristic	Total, N = 313	Appendicular perforation		*p* value
		No, N = 207	Yes, N = 106	
Age (years)				< 0.001
< 4	39 (12.46%)	16 (7.73%)	23 (21.70%)	
4–9	170 (54.31%)	107 (51.69%)	63 (59.43%)	
> 9	104 (33.23%)	84 (40.58%)	20 (18.87%)	
Sex				0.126
Male	184 (58.79%)	128 (61.84%)	56 (52.83%)	
Female	129 (41.21%)	79 (38.16%)	50 (47.17%)	
Body temp. (°C)				< 0.001
< 37.4	149 (47.60%)	128 (61.84%)	21 (19.81%)	
37.4–38.5	71 (22.68%)	43 (20.77%)	28 (26.42%)	
> 38.5	93 (29.71%)	36 (17.39%)	57 (53.77%)	
Symptom duration (h)	24 (18–72)	24 (13–48)	48 (24–96)	< 0.001
< 24	89 (28.43%)	81 (39.13%)	8 (7.55%)	
24–72	133 (42.49%)	87 (42.03%)	46 (43.40%)	
> 72	91 (29.07%)	39 (18.84%)	52 (49.06%)	
WBC (× 10^9^/L)	14 (10.4–17.4)	13.5 (9.3–16.8)	14.7 (11.7–18.4)	0.006
Neutrophils (%)	82.1 (71.8–88.2)	81 (68.1–86.6)	84.15 (77.1–89.5)	0.002
Lymphocytes (%)	11.4 (7–19.5)	13.2 (7.7–24)	9.75 (6.3–14.7)	< 0.001
Hemoglobin (g/L)	130 (123–136)	131 (123–136)	129 (118–135)	0.052
Platelets (× 10^9^/L)	299 (255–355)	297 (254–345)	315 (257–389)	0.05
C-reactive protein (mg/L)	26.44 (6.72–86.64)	14.3 (3.16–37.28)	88.2 (42.7–153.83)	< 0.001
Procalcitonin (ng/mL)	0.22 (0.05–1.25)	0.07 (0.05–0.32)	1.23 (0.25–5.62)	< 0.001
Total bilirubin (μmol/L)	10.8 (8.1–15.1)	10.5 (8–14)	11.9 (8.2–17.4)	0.046
Direct bilirubin (μmol/L)	3.3 (2.3–5)	3.1 (2.2–4.6)	3.8 (2.6–6.1)	0.005
Albumin (g/L)	44.9 (42.6–46.7)	45.1 (43.7–47.1)	43.3 (40.9–45.9)	< 0.001
Prealbumin (mg/dL)	18 (13.6–22.6)	20.2 (16.7–24.9)	13.6 (10.1–16.5)	< 0.001
Alanine aminotransferase (U/L)	13 (10–17)	13 (10–17)	12 (9–15)	0.084
Aspartate aminotransferase (U/L)	25 (21–32)	26 (21–33)	24 (21–31)	0.506
Serum potassium (mmol/L)	4.07 (3.83–4.38)	4.1 (3.85–4.38)	4.04 (3.82–4.39)	0.546
Serum sodium (mmol/L)	138 (136–139)	138 (137–140)	136 (133–138)	< 0.001
Serum chlorine (mmol/L)	102 (99–104.7)	103 (100–105.1)	99.95 (96–102.4)	< 0.001
NLR	7.23 (3.67–12.63)	6.21 (2.83–11.2)	8.6 (5.14–14.08)	< 0.001
PLR	1.93 (1.23–3.07)	1.72 (1.14–2.85)	2.46 (1.56–3.44)	< 0.001
CPA	1.45 (0.31–5.88)	0.7 (0.11–2.18)	6.63 (2.9–13.02)	< 0.001

Data is expressed in median and IQR.

*NLR* neutrophil-to-lymphocyte ratio, *PLR* platelet-to-lymphocyte ratio, *CPA* C-reactive protein-to-prealbumin ratio.

Univariate analysis indicated that age, body temperature, symptom duration, CRP, and other factors pose risks for perforated appendicitis. Multivariate analysis identified symptom duration (OR 2.68, 95% CI 1.570–4.587, *p* < 0.001), PLR (OR 2.75, 95% CI 1.135–6.663, *p* = 0.025), and CPA (OR 5.55, 95% CI 1.729–17.83, *p* = 0.004) as independent risk factors for perforated appendicitis, as detailed in Table 2.

**Table 2:** Risk factors for perforated appendicitis.

Variable	Univariate analysis			Multivariate analysis		
	OR	95% CI	*p* value	Adjust OR	95% CI	*p* value
Age	0.407	0.275–0.602	< 0.001	0.639	0.367–1.115	0.115
Body temp	3.088	2.271–4.199	< 0.001	1.438	0.933–2.218	0.1
Symptom duration	3.343	2.315–4.828	< 0.001	2.684	1.570–4.587	< 0.001
WBC	1.538	0.960–2.464	0.074			
Neutrophils	1.567	0.977–2.513	0.062			
Lymphocytes	0.535	0.333–0.860	0.01	0.413	0.034–4.989	0.486
C-reactive protein	9.778	5.455–17.53	< 0.001	1.042	0.327–3.320	0.944
Procalcitonin	3.733	2.105–6.622	< 0.001	1.235	0.551–2.769	0.608
Total bilirubin	1.373	0.850–2.215	0.195			
Direct bilirubin	1.925	1.185–3.129	0.008	1.363	0.670–2.774	0.393
Albumin	0.359	0.221–0.583	< 0.001	0.975	0.459–2.067	0.946
prealbumin	0.152	0.088–0.263	< 0.001	0.732	0.334–1.602	0.435
Serum sodium	0.270	0.156–0.467	< 0.001	0.814	0.387–1.714	0.589
Serum chlorine	0.273	0.164–0.454	< 0.001	0.794	0.387–1.629	0.53
NLR	1.761	1.096–2.831	0.019	0.599	0.051–6.982	0.683
PLR	2.921	1.785–4.780	< 0.001	2.749	1.135–6.663	0.025
CPA	14.833	8.104–27.15	< 0.001	5.552	1.729–17.83	0.004

Symptom duration, PLR, and CPA were identified as independent risk factors for perforated appendicitis in the multivariate analysis.

*NLR* neutrophil-to-lymphocyte ratio, *PLR* platelet-to-lymphocyte ratio, *CPA* C-reactive protein-to-prealbumin ratio, *OR* odds ratio, *CI* confidence interval.

### Subgroup analysis

In children below 4 years of age, the perforated appendicitis group exhibits lower levels of hemoglobin, albumin, and prealbumin compared to the non-perforated appendicitis group (*p* < 0.05). However, the differences in the remaining indicators were not significant between the non-perforated and perforated appendicitis groups (*p* > 0.05).

For children aged 4–9 years, there were significant differences in body temperature, symptom duration, white blood cells, neutrophils, lymphocytes, platelets, CRP, procalcitonin, direct bilirubin, albumin, prealbumin, and other indicators between the perforated and non-perforated groups (*p* < 0.05). Specifically, the median CPA of the perforated appendicitis group was 7.53 (2.97–12.44), while that of the non-perforated appendicitis group was 0.83 (0.3–2.41), showing a significant distinction (*p* < 0.001).

In the 9 to 16-year-old age group, there was no significant difference in hemoglobin between the non-perforated and perforated groups (*p* > 0.05). However, notable variations existed in other indicators (*p* < 0.001). Particularly, the median CPA for the perforated appendicitis group stood at 8.12 (2.99–15.17), significantly higher than that of the non-perforated appendicitis group (0.62; 0.11–1.8), with a marked significant (*p* < 0.001). Refer to Table 3 for detailed insights into these findings.

**Table 3:** Perforated and non-perforated appendicitis data by subgroup.

Characteristic	Age ≤ 4, N = 39		*p *value	4 < age ≤ 9, N = 170		*p *value	Age > 9, N = 104		*p *value	*p *value^¶^
	Appendicular perforation			Appendicular perforation			Appendicular perforation			
	No, N = 16	Yes, N = 23		No, N = 107	Yes, N = 63		No, N = 84	Yes, N = 20		
Body temp. (°C)			0.813*			< 0.001			< 0.001*	< 0.001
< 37.4	2 (12.5%)	4 (17.39%)		61 (57.01%)	11 (17.46%)		65 (77.38%)	6 (30%)		
37.4–38.5	5 (31.25%)	5 (21.74%)		25 (23.36%)	16 (25.4%)		13 (15.48%)	7 (35%)		
> 38.5	9 (56.25%)	14 (60.87%)		21 (19.63%)	36 (57.14%)		6 (7.14%)	7 (35%)		
Symptom duration (h)			0.152*			< 0.001			0.001	< 0.001
< 24	5 (31.25%)	2 (8.7%)		38 (35.51%)	5 (7.94%)		38 (45.24%)	1 (5%)		
24–72	4 (25%)	5 (21.74%)		55 (51.4%)	31 (49.21%)		28 (33.33%)	10 (50%)		
> 72	7 (43.03%)	16 (69.57)		14 (13.08%)	27 (42.86%)		18 (21.43%)	9 (45%)		
WBC (× 10^9^/L)	14 (11–21)	13 (9.4–17.7)	0.416	14.2 (10.6–16.8)	15 (12.7–18.4)	0.022	12.25 (8.05–16.25)	14.85 (12.2–18.75)	0.048	0.006
Neutrophils (%)	83 (72–86)	73.7 (70.3–86.9)	0.578	81.2 (73–86.6)	85.8 (78.3–89.6)	0.002	79.85 (62.35–87.3)	85.75 (80.25–91.5)	0.011	< 0.001
Lymphocytes (%)	11 (9–20)	17.1 (8–22.3)	0.493	13.1 (7.8–19.5)	8.5 (6–13)	< 0.001	14.2 (7.55–29.6)	8.25 (5.05–12.65)	0.011	< 0.001
Hemoglobin (g/L)	127 (121–134)	118 (111–126)	0.038	128 (122–135)	129 (122–134)	0.811	133 (128.5–140.5)	136 (125–143.5)	0.509	0.806
Platelets (× 10^9^/L)	304 (237–358)	292 (214–405)	0.658	291 (254–345)	340 (281–393)	0.003	298.5 (252.5–345)	277 (238–347.5)	0.382	0.015
C-reactive protein (mg/L)	64 (8–102)	111.76 (33.72–161.68)	0.084	16.79 (6.48–43.31)	86.64 (42.7–154.11)	< 0.001	7.1 (1.38–23.59)	88.2 (48.22–143.36)	< 0.001	< 0.001
Procalcitonin (ng/mL)	0 (0–1)	1.25 (0.25–5.12)	0.127	0.16 (0.05–0.58)	1.86 (0.23–6.85)	< 0.001	0.05 (0.05–0.16)	0.38 (0.24–0.93)	0.004	< 0.001
Total bilirubin (μmol/L)	9 (6–13)	9.2 (6.6–16.2)	0.627	10.2 (8.2–13.1)	11.7 (8.1–15.6)	0.313	11.2 (8.05–15.2)	17.1 (14.55–19.6)	< 0.001	0.018
Direct bilirubin (μmol/L)	3 (2–4)	3.2 (2–4.3)	0.291	3 (2.2–4.4)	3.8 (2.7–5.6)	0.018	3.35 (2.45–5.3)	4.85 (4.15–7.8)	0.004	< 0.001
Albumin (g/L)	45 (43–46)	41.5 (39.7–45.3)	0.019	45 (43.8–47)	43.2 (40.3–46.2)	< 0.001	45.7 (43.5–47.2)	45.35 (41.55–46.25)	0.135	< 0.001
Prealbumin (mg/dL)	14 (12–19)	9.95 (8.1–13.1)	0.002	18.5 (15.6–21.9)	14.2 (10.5–16.8)	< 0.001	23.35 (19.75–27.2)	14.45 (11.8–20.9)	< 0.001	< 0.001
Alanine aminotransferase (U/L)	13 (11–16)	13 (11–20)	0.43	13 (10–18)	12 (8–15)	0.017	12 (9–17)	11 (10.5–13)	0.315	0.012
Aspartate aminotransferase (U/L)	32 (27–40)	32 (24–50)	0.989	29 (24–34)	25 (22–30)	0.007	21 (19–26)	19.5 (17–22)	0.108	0.002
Serum potassium (mmol/L)	4 (4–5)	4.48 (3.92–4.7)	0.558	4.11 (3.9–4.45)	4.01 (3.8–4.3)	0.06	3.99 (3.8–4.2)	3.91 (3.68–4.13)	0.183	0.03
Serum sodium (mmol/L)	136 (134–137)	136 (133–139)	0.763	138 (136–139)	136 (134–138)	< 0.001	139 (138–140)	135.5 (133–138)	< 0.001	< 0.001
Serum chlorine (mmol/L)	100 (98–102)	100.8 (97–102.8)	0.648	103 (100–105.4)	99 (96–102)	< 0.001	103.8 (101–105.65)	100.75 (98–103.5)	0.003	< 0.001
NLR	8 (4–9)	4.16 (3.15–10.51)	0.521	6.25 (3.71–10.83)	10.33 (6.02–14.73)	< 0.001	5.55 (2.14–11.67)	10.5 (6.33–17.98)	0.01	< 0.001
PLR	2 (1–2)	1.67 (1–2.81)	0.898	1.65 (1.12–2.68)	2.76 (1.96–3.47)	< 0.001	1.75 (1.14–2.9)	2.5 (1.62–4.38)	0.029	< 0.001
CPA	4 (0–7)	6.85 (2.37–19.48)	0.053	0.83 (0.3–2.41)	7.53 (2.97–12.44)	< 0.001	0.32 (0.05–0.99)	4.62 (2.66–12.95)	< 0.001	< 0.001

The data is expressed in median and IQR.

*NLR* neutrophil-to-lymphocyte ratio, *PLR* platelet-to-lymphocyte ratio, *CPA* C-reactive protein-to-prealbumin ratio.

*The application of Fisher's exact test.

^¶^Significant differences between the perforated appendicitis and non-perforated appendicitis subgroups, determined through the Friedman test.

**Figure 3:**
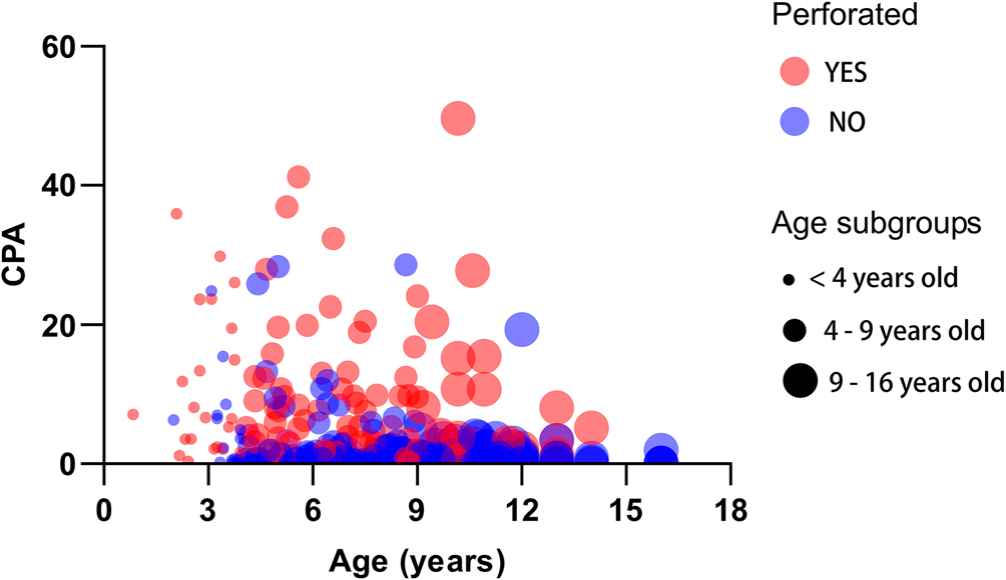
CPA distribution in children with non-perforated appendicitis and perforated appendicitis by age group. The graph illustrated the CPA distribution in children with non-perforated appendicitis and perforated appendicitis across age subgroups. In children below 4 years of age, the CPA of perforated appendicitis patients was not significantly different from that of the non-perforated appendicitis patients [6.85 (2.37–19.48) vs. 4 (0–7), *p* = 0.053]. In children aged 4–9 years and over 9 years, the CPA for perforated appendicitis was significantly higher than that for non-perforated appendicitis [7.53 (2.97–12.44) vs. 0.83 (0.3–2.41), *p* < 0.001] [4.62 (2.66–12.95) vs. 0.32 (0.05–0.99), *p* < 0.001]. *CPA* C-reactive protein-to-prealbumin ratio.

Figure 3 illustrates the distribution of CPA among both the non-perforated and perforated patients within each subgroup.

### ROC curve analysis

**Figure 4:**
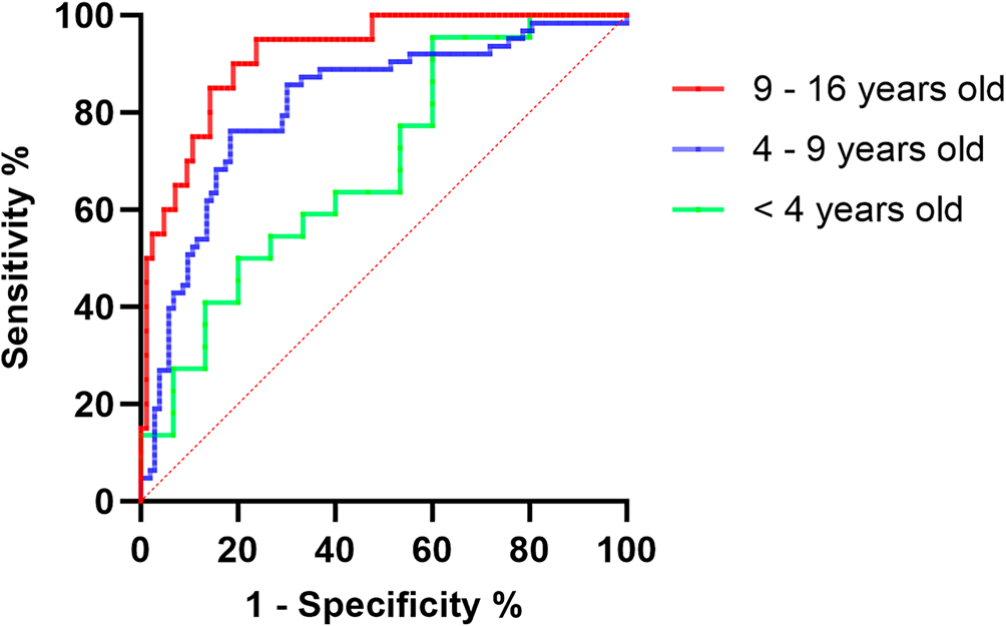
ROC curve of predicting perforated appendicitis in children based on CPA. In children below 4 years of age, the AUC of CPA was 0.691 (95% CI 0.513–0.869, *p* = 0.084). For children aged 4–9 years, the AUC of CPA increased to 0.816 (95% CI 0.747–0.886, *p* < 0.001). For children aged 9–16 years, the AUC of CPA further improved to 0.919 (95% CI 0.859–0.979, *p* < 0.001). Consequently, the accuracy of predicting perforated appendicitis based on CPA demonstrates an increasing trend with the age of the children. *CPA* C-reactive protein-to-prealbumin ratio, *AUC* area under the curve.

The ROC curve analysis resulted in an AUC for CPA of 0.691 (95% CI 0.513–0.869, *p* = 0.084) for children below 4 years old. In the 4 to 9-year-old group, the AUC for CPA was 0.816 (95% CI 0.747–0.886, *p* < 0.001), indicating an optimal cut-off of 3.0, a sensitivity of 76.2%, and a specificity of 81.6%. Among children aged 9 to 16 years, the AUC for CPA was 0.919 (95% CI 0.859–0.979, *p* < 0.001), presenting an optimal cut-off of 2.2, a sensitivity of 85%, and a specificity of 85.7%. Details encompassing the ROC curve, cut-off, sensitivity, and specificity are outlined in Table 4. The accuracy in predicting perforated appendicitis in children over 4 years old via CPA appears notably high, as depicted in in Fig. 4.

**Table 4:** CPA cut-off values for predicting perforated appendicitis by age group.

Cut-off	Sensitivity	Specificity	Positive predictive value	Negative predictive value	Accuracy	Cut-off calculation method
4–9 years old						
2.965	0.762	0.816	0.716	0.848	0.795	Y
2.965	0.762	0.816	0.716	0.848	0.795	C
2.965	0.762	0.816	0.716	0.848	0.795	D
2.57	0.762	0.757	0.658	0.839	0.759	S
9–16 years old						
1.049	0.95	0.762	0.487	0.985	0.798	Y
4.093	0.55	0.976	0.846	0.901	0.894	C
5.14	0.5	0.988	0.909	0.892	0.894	C
2.236	0.85	0.857	0.586	0.96	0.856	D
2.184	0.85	0.845	0.567	0.959	0.846	S

The cut-off values of the ROC curves aimed at predicting perforated appendicitis based on the CPA in children aged 4–9 years and those above 9 years were calculated based on the Youden's index (Y), the maximum accuracy (C), the shortest distance in the upper left corner of the ROC curve (D), and the smallest difference between sensitivity and specificity absolute values (S), respectively. In this particular investigation, the D method was chosen to ascertain the optimal cut-off values.

*CPA* C-reactive protein-to-prealbumin ratio.

## Discussion

In prior investigations on serum biomarkers, various factors such as NLR, PLR, WBC, lymphocyte ratio, CRP, erythrocyte sedimentation rate, elevated serum total bilirubin levels, and decreased serum sodium levels have been identified as potential predictors of perforated appendicitis^8–13,15^. In contrast, this study proposes PLR and CPA as independent risk factors for perforated appendicitis.

The study also highlights CPA as a valuable marker for predicting perforated appendicitis in children. CRP, a commonly used marker for inflammatory response, typically rises 4–6 h after infection onset, peaks at 36–72 h, and gradually decreases 4 h after the infection is controlled. This makes it a marker to assess the inflammatory response in acute appendicitis^16^. Prealbumin, an unglycosylated plasma protein synthesized in the liver, is frequently employed to assess recent nutritional status and is considered a “negative acute phase reactant,” reflecting the severity of various acute inflammatory diseases^17^. During perforated appendicitis, the liver's synthesis of prealbumin decreases due to poor protein absorption caused by inflammation, resulting in decreased prealbumin levels^14^. Combined with the aforementioned factors, CPA can provide a more comprehensive reflection of the patient's physiological state of the patient. ROC curve analysis indicates that in children aged 4–9 years, a CPA value greater than 3 serves as a predictor of perforated appendicitis. With an AUC of 0.816, the diagnostic value is high. In children aged 9–16 years, a CPA value greater than 2.2 can effectively predict perforated appendicitis, boasting a high AUC of 0.919, signifying exceptional diagnostic accuracy. Subgroup analysis reveals a significant CPA difference between the perforated and non-perforated groups in children aged above 4 years old. However, in children below 4 years old, CPA may not be applicable for predicting perforated appendicitis, possibly due to their inadequate immune system development and poor response to inflammatory stimuli^18–21^. These changes underscore the impact of age-related differences in immune system development and physiological characteristics among children^22^.

A META-analysis identifies NLR as a predictor for pediatric acute perforated appendicitis ^8^ (AUC: 0.86, sensitivity: 82%, specificity: 76%). In a cross-sectional study, both NLR (AUC: 0.74, sensitivity: 77.78%, specificity: 67.14%) and PLR (AUC: 0.74, sensitivity: 77.78%, specificity: 63.57%) serve as predictors ^9^. Additionally, a cohort study highlights serum total bilirubin as a potential predictor ^10^ (AUC: 0.876, sensitivity: 92%, specificity: 77.3%). In comparison to prior research, this study emphasizes the robust predictive value of CPA in pediatric acute perforated appendicitis, particularly in older children, exhibiting higher AUC, sensitivity, and specificity.

This study employed subgroup analysis, clearly delineating the characteristics of perforated appendicitis across different age groups. However, due to limitations in sample size, creating too many subgroups would compromise statistical power, leading to the classification of patients into three subgroups. This study excluded patients who received antibiotics before admission, minimizing the impact of antibiotics on changes in inflammatory biomarkers. However, the study has certain limitations. Firstly, the cross-sectional study design complicates the control of pre-admission factors such as medication and diet. Despite efforts to mitigate interfering factors like age, sex, and pre-admission antibiotic use, the results may still be influenced by other unknown factors. Secondly, the relatively small sample size, particularly in the below-4-year-old group, may yield erratic results impacting interpretation and generalization. Additionally, significant differences in fever, platelets, hemoglobin, and other indicators among different subgroups prompt further reflections on changes in patients’ physiological and metabolic states. The higher body temperatures may suggest distinct infection-induced inflammatory processes in the immune system of younger patients compared to older patients,although the effects of other unknown factors cannot be ruled out. It's important to note that the study was conducted within a specific medical institution, limiting the generalizability of findings to other regions. Future longitudinal and multicenter studies could further validate these findings, considering variations across regions, healthcare facilities, and populations.

In clinical practice, this study revealed that CPA offers significant predictive value for perforated appendicitis in children aged over 4 years. Importantly, both CRP and prealbumin levels are easily measurable in various medical institutions at relatively low costs. Moreover, the utilization of CPA aids in the early diagnosis of perforated appendicitis, enabling prompt intervention and perioperative management, ultimately contributing to patient safety during and after surgery and promoting a positive prognosis.

In summary, CPA values exceeding 3 and 2.2 serve as predictors of perforated appendicitis in children aged 4–9 years and 9–16 years, respectively. CPA proves to be a valuable tool in effectively discerning the risk of perforation in children with acute appendicitis, offering crucial insights for early clinical intervention.

## Data Availability

The datasets analysed during the current study are available from the corresponding author upon reasonable request.
